# Perineal skin recurrence on the site of Lone Star Retractor: case report

**DOI:** 10.1186/s40792-017-0405-0

**Published:** 2017-12-28

**Authors:** Mohamed Hamid, Anass Mohamed Majbar, Abdelmalek Hrora, Mohamed Ahallat

**Affiliations:** Faculty of Medicine and Pharmacy of Rabat, Department of Surgery, Ibn Sina Hospital, Mohammed V University of Rabat, Rabat, Morocco

**Keywords:** Neoplasm seeding, Neoplasm recurrence, Rectal neoplasms, Lone Star Retractor, Skin recurrence

## Abstract

**Background:**

Local recurrence of colorectal cancer is a major cause of morbidity and mortality that usually implies a worse prognosis. Its etiopathogenesis is still a subject of debate. Recurrence on the perineal wound caused by anal retractor device is rarely reported.

**Case presentation:**

We present the case of a 75-year-old woman with perineal skin recurrence on the site of Lone Star Retractor™ from rectal adenocarcinoma. The patient underwent a curative proctectomy followed by a hand-sewn coloanal anastomosis using Lone Star Retractor™ 2 years ago for a tumor of the lower rectum. The recurrence was most likely caused by the seeding of exfoliated tumor cells into the perianal skin which was abraded by the retractor.

**Conclusion:**

This case is the fourth case reported in the literature and highlights the importance of the use of less traumatic endoanal retractors to prevent such perianal recurrence. Recurrence on the perineal wound caused by anal retractor device is rare but possible. Further studies are needed to define preventive measures able to reduce cutaneous implants.

## Background

Local recurrence (LR) of colorectal cancer is a major cause of morbidity and mortality that usually implies a worse prognosis. The etiopathogenesis of LR is still a subject of debate, and this has led to major improvements in colorectal management [1–3]. After a curative resection of rectal cancer, LR is often the consequence of inadequate clearance of the tumor or the surrounding tissues; however, another mechanism to explain recurrence on preexisting benign perineal lesions is suggested by Guiss in 1954 [4] who reported the first a case of implantation of cancer cells within a fistula-in-ano. Since this case, several case reports and small case series reported CCR recurrences on preexisting benign perineal lesions such as hemorrhoids [5–8], fistula [9–12], or on the anal wound caused by stapling device [13–15].

LR on the perineal wound caused by anal retractor device is rarely reported, with only three cases reported so far, one case on the scar of a Gelpi Retractor [16] and two on the site of Lone Star Retractor™ [17].

Herein, we report the fourth case of cutaneous perineal recurrence on the site of a Lone Star Retractor ™ system after a curative proctectomy and hand-sewn coloanal anastomosis for rectal cancer.

## Case presentation

**Fig. 1:**
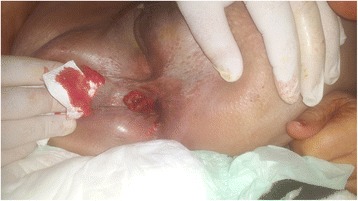
A budding lesion 2 cm on the right of the anal verge, developing from the perianal skin, without continuity with the coloanal anastomosis

**Fig. 2:**
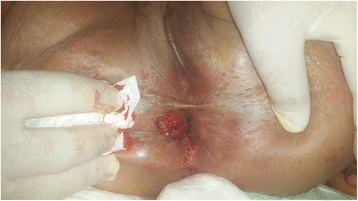
A budding lesion 2 cm on the right of the anal verge, developing from the perianal skin, without continuity with the coloanal anastomosis

A 75-year-old woman presented with a left hemicircumferential adenocarcinoma of the rectum located 4 cm above the anal verge. Her past medical history included type 2 diabetes and hypertension. Her medical history was negative for perineal or anal diseases such fistula or hemorrhoids. The tumor was classified preoperatively as T3 according to the pelvic magnetic resonance imaging (MRI) and endorectal ultrasound. On preoperative workup imaging, there was no evidence of distant metastases. Six weeks after a neoadjuvant chemotherapy, the patient underwent a laparoscopic proctectomy with total mesorectal excision (TME) and intersphincteric resection. The Lone Star Retractor™ was used for the exposure of anal verge, and the perineal dissection was performed transanally. The anal canal was washed with a povidone-iodine solution, and hand-sewn side-to-end coloanal anastomosis was created. A diverting lateral ileostomy was made. The immediate postoperative course was marked by the occurrence of a grade B anastomotic fistula, treated by a transanal drainage system. Pathologic examination of the specimen showed a well-differentiated adenocarcinoma ypT3N0 (12N−/12N) with a poor therapeutic response Dworak 1. The mesorectum was complete, and the circumferential resection margin was 5 mm; the distal margin was 6 mm. The diverting stoma was closed 4 months postoperatively. No adjuvant chemotherapy was administered. On the fifth week postoperatively, the patient presented an acute dehydration with functional renal insufficiency complicated by hyperkalemia leading to cardiac arrest, which was resuscitated and recovered without visceral damage. At 14-month follow-up, the patient presented in poor general condition, with rectal bleeding and right femoropopliteal venous thrombosis. Clinical examination showed a 2-cm budding lesion on the right of the anal verge, developing from the perianal skin, 1 cm from the anal border without continuity with the coloanal anastomosis; the location of the lesion corresponded to the insertion site of one of the elastic hooks of the Lone Star Retractor (Figs. 1 and 2). Digital examination founded a local recurrence. At the CT, there was no distant recurrence. The histologic biopsy showed a well-differentiated adenocarcinoma, similar to that of the original specimen. The patient was not operated because of her general condition. No further treatment was given.

## Discussion

Local recurrence is a major cause of morbidity and mortality, usually implies a worse prognosis. It may occur as a result from two potential etiopathogeneses. First, metastases through endothelial-lined channels occur to both lymphatic and hematogenous routes [18], which are controlled by neoadjuvant therapy and optimal TME. Indeed, TME reduced the high local recurrence rates from 30–40% to 5–10% [19] and even greater when associated with chemoradiotherapy (CRT) [20]. The second is by implantation of viable exfoliated malignant cells from a rectal adenocarcinoma on preexisting benign perineal lesions [21–24]; it was estimated that 70% of the specimens were found to have viable exfoliated colorectal cancer cells with median cell number ranging from 0.55 × 105 to 0.78 × 10 [6].

Since the first case of cell implantation into the anal fistula in 1954 by Guiss [4], several case reports and small case series reported the colorectal cancer recurrence on preexisting benign perineal lesions such as hemorrhoid [5–8], fistula [9–12], or on the anal wound caused by stapling device manipulation [2, 13–15]. In this case, the surgery was considered to be “curative” R0 resection; the mesorectum was complete, and the pathologic finding was tumor-free with adequate margins, but malignant cells had probably implanted into the perianal skin wound caused by the Lone Star Retractor hooks. Tranchart [17] and Cantos-Pallares [25] have reported three recurrence cases after using the same retractor. Zinzindohoue [16] reported a case of tumor recurrence on the scar of a Gelpi Retractor.

We propose, as Tranchart [17] did, the use of less traumatic endoanal retractors to prevent such perianal recurrence. Using intraoperative rectal washout with cytocidal solutions is usually recommended to reduce the amount and viability of malignant cells, a procedure that reduces the LR risk after anterior resection. Several studies have highlighted on the impact of washout on the LR rate after anterior resection with conflicting results [26]. However, in both patients of Tranchart [17], and in this case, recurrence occurred despite preoperative rectal washing.

The management of colorectal cancer LR is still a matter of debate, it ranges from local excision [15–17] to a more aggressive approach [25, 27]. A local excision in case of cutaneous perianal metastases seems to be adapted because implantation of exfoliated tumor cells is distal to the anastomosis and not associated to pelvic recurrence [17]. The previously reported LR on the site of retractor [16, 17, 25] was locally excised with wide margins, and no further adjuvant treatment was given. These patients did not show any other signs of recurrence a year later.

## Conclusion

Local recurrence of colorectal cancer is a major cause of morbidity and mortality that usually implies a worse prognosis. Recurrence on the perineal wound caused by anal retractor device is rare but possible. The knowledge of this mechanism of occurrence may change surgical practices and push for further studies to define preventive measures that can reduce skin implants.
